# Evaluation of the benefit of using blue dye in addition to indocyanine green fluorescence for sentinel lymph node biopsy in patients with breast cancer

**DOI:** 10.1186/1477-7819-12-290

**Published:** 2014-09-20

**Authors:** Wenbin Guo, Li Zhang, Jun Ji, Wei Gao, Jintao Liu, Meng Tong

**Affiliations:** The Breast Centre, Department of Surgery, Dalian Central Hospital, Dalian Medical University, 826, Xinan Road, Shahekou District, Dalian 116033 China; The Central Laboratory, Dalian Central Hospital, Dalian Medical University, 826, Xinan Road, Shahekou District, Dalian 116033 China

**Keywords:** Sentinel lymph node biopsy, Breast cancer, Indocyanine green, Fluorescence imaging, Blue dye

## Abstract

**Background:**

Near infrared-guided indocyanine green (ICG) fluorescence has vast potential for guiding sentinel lymph node biopsy (SLNB) in patients with breast cancer. The purpose of this study was to evaluate any additional clinical benefit for SLNB when blue dye is used in combination with ICG.

**Methods:**

Between November of 2009 and September of 2013, 86 patients diagnosed with breast cancer were investigated by SLNB using a combination of patent blue and ICG. A lymph node was considered as the sentinel lymph node (SLN) when it was stained with blue dye and/or fluorescence. A levelIandIIaxillary dissection was performed for verification of axillary node status after the SLNB.

**Results:**

The SLN identification rate of SLN for ICG-patent blue combination was comparable to that for ICG alone (98.8% versus 93%; *P* = 0.054), but the false-negative rate was reduced from 12% (3/25) to 4% (1/25). Twenty-four patients had positive SLNs. In two of those patients, although there were SLNs identified by both tracers, the positive SLNs were identified by blue dye only.

**Conclusion:**

Although blue dye did not improve the identification rate significantly, there was a definite benefit in improving the false-negative rate. The use of a fluorescence method together with blue dye is an ideal method for hospitals that do not have access to conventional radiation-based detection methods.

## Background

Axillary lymph node status has consistently been shown to be the most significant prognostic factor in patients with breast cancer [1–4]. The method of sentinel lymph node biopsy (SLNB) is now well accepted for evaluation of axillary node status.

Large validated studies including the ALMANAC trials have shown that SLNB in patients with breast cancer is a safe, reliable technique that stages the axilla accurately [5, 6].

SLNB has been performed using different techniques: injection of blue dye, radioactive colloid or the combination of both. Although high rates of sentinel lymph node (SLN) detection have been obtained with all of these methods, there is no general consensus about the optimal technique [7, 8]. The average rate of SLN identification using blue dye or radioactive colloid is over 90%, but ranges from 65 to 98% [9]. Some surgeons use the blue dye method alone. Although the dye method has several benefits including ease of use, cost effectiveness and safety, it has been pointed out that the detection rate is lower than that of the gamma probe method, and combined mapping with radiocolloid and blue dye has been shown to be superior to blue dye alone [7].

Preoperative lymphoscintigraphy facilitates intraoperative identification of axillary nodes, but there are concerns about limited availability and cost of radiocolloids, and the exposure of healthcare professionals to radiation [10, 11]. However, lymphoscintigraphy with a radioisotope cannot clearly visualize the lymphatic drainage pathway.

Some studies have reported that indocyanine green (ICG) could be used for SLNB in breast cancer [11, 12]. The photodynamic eye (PDE) can visualize the lymphatic drainage pathway clearly and demonstrate the accurate location of SLN real-time in the operating room [13]. However, little is known to date about SLNB by combining ICG with other tracers in breast cancer.

The aim of this study is to evaluate whether there is an additional benefit to using blue dye in addition to ICG fluorescence in breast cancer, to provide a foundation for improvement of the SLNB method.

## Methods

### Patients

Eighty-six consecutive patients diagnosed with breast cancer between November 2009 and September 2013 underwent SLNB using a combination of patent blue and ICG at the Breast Centre of Dalian Central Hospital. All patients were women with early stage breast cancer. Exclusion criteria included: palpable axillary lymph nodes, tumour diameter > 3 cm, multicentric tumour, previous breast or axillary surgery, pregnancy, and allergy to iodine or shellfish. Written consent was obtained from all participants, and the study was approved by the institutional ethics committee of Dalian Central Hospital.

### Operative procedure

**Figure 1:**
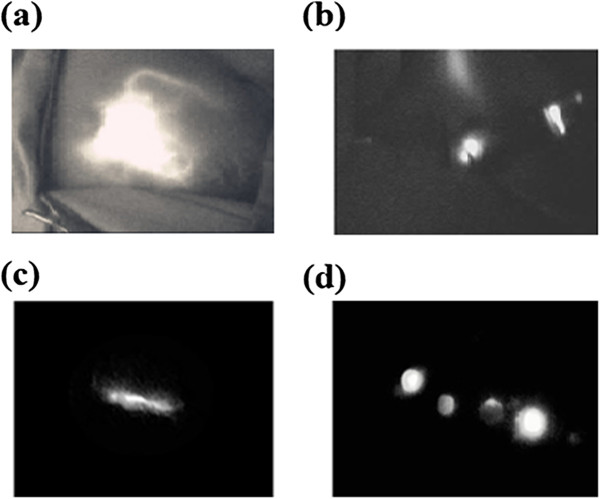
**Sentinel lymph node biopsy (SLNB) using indocyanine green fluorescence imaging: (a)** The periareolar injection site and afferent lymphatic duct were clearly observed. **(b)** A skin incision was made at the point where the fluorescence disappeared and a strong fluorescence could be seen easily after incision. **(c)** A lymphatic flow in the axilla was identified. **(d)** The resected SLNs with fluorescence imaging were reconfirmed by the photodynamic eye (PDE).

SLNB was performed before wide excision, breast conserving surgery, or mastectomy as follows. Under anaesthesia, 2 mL patent blue (Bleu Patente V, Guerbet, Brussels, Belgium) was injected into the subareolar region and skin overlying the tumour. The whole breast was massaged for about 5 minutes, then 1 mL of 1:20 diluted ICG solution (Diagnogreen, Daiichi Pharmaceuticals, Tokyo, Japan) was injected into the subareolar region followed by a 30-second breast massage to facilitate the absorption of ICG into the lymph vessels. At the end of the massage, the surgical lights were turned off. Image acquisition was performed under near-infrared light (NIR, λ = 760 nM) using a photodynamic eye (Hamamatsu City, Japan). The fluorescence emitted by ICG was followed in the direction of the areola towards the axilla, allowing visualisation of the subcutaneous lymphatic drainage pathway. A marking was made on the skin where the fluorescent signal disappeared, and a skin incision was made at that point.After the subdermal layer was removed, the lymphatic duct identified by ICG was visible. To avoid lymphatic fluid retention, the main lymphatic duct was ligated at a site proximal to the first SLN, because ligating the afferent lymphatic vessel prevents ICG from accumulating in the operative field. At this point, further fluorescent imaging was performed to identify any sites that emitted a strong fluorescence, and such sites were removed along with the surrounding tissue. Following ICG-assisted dissection, the surgical lights were turned on and blue-stained nodes were excised under the naked eye. All fluorescent and/or blue-stained SLNs were harvested (Figure 1). A standard level I and II axillary lymph node dissection (ALND) was performed for verification of axillary node status after SLNB. Immediately after harvesting, SLNs were sent to the pathology department for intraoperative frozen section evaluation. All SLNs were histopathologically evaluated by 3-mm sectioning and staining with H&E. All SLNB procedures were performed or supervised by two senior breast surgeons. Formalin-fixed paraffin embedded tissue sections of SLNs and non-SLNs were stained with H&E.

Patient data were entered prospectively into a database and statistically analysed using SPSS software (SPSS, Chicago, IL, USA). A Chi-square test was used to determine statistical significance. A *P*-value of ≤ 0.05 was considered to be statistically significant.

## Results

Patient data are shown in Table 1. The mean age was 52.6 years old (range: 32 to 76 years). The primary tumour was located on the right for 40 cases (46.5%) and on the left for 46 cases (53.5%). None of the 86 patients experienced adverse effects in response to ICG or patent blue. ICG fluorescence imaging and/or blue staining identified at least one SLN in all but one patient whose SLN was not found and her axillary dissection confirmed that there was no positive lymph node. The SLN identification rate was 98.8% (85/86 patients). A total of 291 SLNs were identified: 36 were fluorescent, 10 were blue and 245 were both fluorescent and blue. In 5 patients, SLNs were identified by patent blue alone, and in 15 they were identified by ICG alone. Therefore, if patent blue alone was used for the procedure, the identification rate would have been 81.4% (70/86). In contrast, if ICG alone was used, the identification rate would have been 93.0% (80/86). There was no significant difference between ICG-patent blue combination and ICG alone on SLN identification (98.8% versus 93.0%; *P* = 0.054) (Table 2).

**Table 1:** **Clinical characteristics of the 86 women undergoing surgery for breast cancer**

Characteristic	Number	(%)
Median age (range), years	52.6	(32 to 76)
Tumour classification		
Tis	12	(14.0)
T1	28	(32.6)
T2	46	(53.4)
Tumour location		
Right	40	(46.5)
Left	46	(53.5)
Grade		
1	16	(18.6)
2	48	(55.8)
3	22	(25.6)
Pathological node status		
Negative	61	(70.9)
Positive	25	(29.1)
Hormonal status		
Negative	36	(41.9)
Positive	50	(58.1)
HER2/neu		
Negative	71	(82.6)
Positive	15	(17.4)

HER2/neu: human epidermal growth factor receptor-2.

**Table 2:** **Sentinel lymph node status as determined by fluorescence navigation, blue dye, and a combination of both in 86 patients**

	Fluorescence navigation (%)	Combination of blue dye and fluorescence (%)	***P***
SLN identified	80 (93.0)	85 (98.8)	0.054
Positive lymph node	25 (29.1)	25 (29.1)	
Positive SLN	22 (25.6)	24 (27.9)	
Accuracy	77 of 80 (96.2)	84 of 85 (98.8)	0.283
False-negative rate	3 of 25 (12.0)	1 of 25 (4.0)	
Mean number of SLNs excised	2.4	3.6	

SLN: sentinel lymph node; Chi-square test.

Metastatic involvement of SLNs was identified by ICG-patent blue in 24 of 25 patients with positive lymph nodes. There was one false-negative case. In this patient, we found only one SLN with no metastases, which was both fluorescent and blue, but ALND revealed that there were two positive axillary nodes. In two of 24 patients with positive SLNs, although SLNs were identified with both ICG and/or patent blue, the positive SLNs were identified with blue dye alone. In the first patient in whom two SLNs were found, there was a positive SLN detected by patent blue alone, and there was a negative SLN that was both fluorescent and blue, but ALND of this patient revealed that there were two additional positive lymph nodes. In the second patient, we only detected two SLNs that were negative and both of them were fluorescent and blue, but her ALND demonstrated one positive lymph node.

As mentioned, in 2 of the 24 patients with positive SLNs, the nodes were identified with blue dye only. In contrast, there were three patients whose positive SLNs were identified with ICG only. If blue dye alone was used, the positive lymph nodes would have been missed in 4 patients, resulting in a false-negative rate of 16% (4/25). If ICG was used, the positive lymph nodes would have been missed in 3 patients, resulting in a false-negative rate of 12% (3/25). As described above, a positive SLN for the combination was only missed in a single patient giving a false-negative rate of 4% (1/25). In addition, the average number of SLNs detected in the combined group was high compared to that in the ICG group (3.6 versus 2.4, Table 2).

## Discussion

Lymphography, in preparation for SLNB, has come to the fore as a major field of application in lymphatic imaging for malignant neoplasia with potential lymph nodal spread and has had a significant impact on overall prognosis [14, 15]. SLNB is an accepted method for staging axillary lymph nodes in women with early breast cancer, and different advantages and disadvantages are associated with different tracer methods. Some advocate blue dye alone, others radioisotope (RI) only, and many utilized a combination of both. Some studies have demonstrated improved identification rate and lower false-negative rate using a combination of blue dye and RI [16]. McMasters *et al*. [17] reported an identification rate of 98.0% and a false-negative rate of 6.5% with the combination of RI (dermal injection) and isosulfan blue dye. Currently, two types of tracer, dye and RI, are used to detect SLNs in breast cancer patients, and a combined use of the two tracers has yielded a high diagnostic value. Now, the combination method is a standard technique. However, the use of a radiotracer is associated with significant expense and requires radiation measures, and RI is not generally available. Some clinicians have used blue dye alone to perform SLNB in breast cancer and the SLN identification rate was always about 85% by using the method, suggesting that the lower identification rate was not related to the surgeon’s experience but is intrinsic to the technique [18].

ICG as a tracer has been shown to be safe in over 40 years of clinical usage [19, 20]. Kitai *et al*. [21] reported the use of fluorescent imaging with ICG during SLNB for breast cancer. Fluorescence-guided imaging with ICG involves advantages compared to the conventional methods, including the combination of transcutaneous and *in situ* navigation, real-time lymphography, a low incidence of adverse reactions of ICG as well as a SLNB procedure without radioactive tracers but a high sensitivity [10]. Moreover, a significant risk of anaphylactic reaction from the blue dye could be spared [22].

However, little is known to date about SLNB combined with ICG and other tracers in breast cancer. The purpose of this study was to determine whether there is an added benefit from using blue dye with ICG when performing SLNS in breast cancer. In this study, all SLNs were identified exclusively by bright fluorescence and/or dye labeling (Figure 1). ICG-guided SLNs resulted in a detection rate of 93.0%, and the identification rate of SLNs using ICG with blue dye was 98.8%, similar to or better than those reported by other researchers, which were obtained by the gamma probe or the combined method that used the gamma probe and the dye [23]. There was no obvious difference in the SLN identification rate between ICG alone and ICG in combination with patent blue (*P* = 0.054) (Table 2). The SLN identification rate was acceptable using either ICG or using a combination of agents. In one patient out of eighty-six cases without any SLN detected, a faint track to mediastinal lymph nodes but without a clear path to the axilla was observed. When axillary dissection was performed, we could not find any fluorescent and/or blue-stained lymph nodes in the patient and there were no positive lymph nodes. In this study, lymph nodes existing outside the axilla were not examined and because the patient was a case with an internal mammary route, we did not find the SLN in the axilla.

McMasters *et al*. [17] also found that the dual-agent technique resulted in a lower false-negative rate compared to the single agent that was used in their study. In the present study, when SLNB was guided by only the fluorescent method, the false-negative rate was 12%, which created a risk of missing appropriate SLNs in the patients. However, by the combination method it was 4%. A false-negative rate of greater than 5% is unacceptable, and thus, even though the blue dye only improved the identification rate by 5%, the false-negative rate was reduced from 12% to the clinically acceptable rate of 4%. Although the false-negative rates would not be statistically different between ICG used alone and the combination method because of the relatively small numbers of false-negative results, there was a trend for improved false-negative rate for using blue dye in addition to ICG fluorescence, which may be clinically meaningful. The results show that there was a higher false-negative rate if the fluorescent technique was used alone, but by using a combination method, it would be acceptable.

In our study, ICG fluorescence with blue dye identified a mean of 3.6 SLNs per patient and the accuracy was 98.8%. In contrast, use of ICG-guided method alone identified only a mean of 2.4 SLNs per patient (Table 2). In the procedure, we tried to remove more SLNs because recent publication has shown that the examination of two nodes provides 91 to 98% accuracy of nodal status, whereas the examination of 4 nodes provides accuracy greater than 98% [24].

There is a learning curve involved in effective performance of SLNB [25, 26]. That is, SLNs can be detected more rapidly and precisely as the surgeon gains more experience of cases requiring SLNB. The blue dye method is helpful in training surgical residents and other surgeons who are learning how to perform the procedure [27]. An advantage of ICG fluorescence is that it enables visualization of lymph flow from the breast to the axilla. SLNs were identified and resected more rapidly and easily by introducing fluorescence and the method was particularly beneficial in difficult cases where SLNs were not readily identified using the dye method [28]. Thus, it can be said that SLNB using ICG fluorescence could be very useful. A disadvantage of ICG fluorescence is that leakage and pollution of ICG causes halation of the image, that is, a glow associated with ICG leakage in the entire surgical field shown in a fluorescence image. When the first SLN is removed and the associated lymphatic ducts have been cut, ICG spreads to the surgical field making it difficult to detect another fluorescent node. This problem can be solved by ligating the main lymphatic duct at a site proximal to the first SLN to avoid lymphatic fluid retention, because ligating the afferent lymphatic vessel prevents ICG from accumulating in the operative field [24].

## Conclusions

The combination of ICG fluorescence with visible patent blue dye is a highly sensitive method for SLN identification during SLNB in breast cancer staging, with a clinically acceptable false-negative rate and no exposure to radiation. The dual-tracer method would be particularly useful in institutions where radiotracers availability is limited.

## Abbreviations

ALND: axillary lymph node dissection
H&E: haematoxylin and eosin
HER2: human epidermal growth factor receptor-2
ICG: indocyanine green
NIR: near-infrared light
PDE: photodynamic eye
RI: radioisotope
SLN: sentinel lymph node
SLNB: sentinel lymph node biopsy.
